# Optimizing RNNs for EMG Signal Classification: A Novel Strategy Using Grey Wolf Optimization

**DOI:** 10.3390/bioengineering11010077

**Published:** 2024-01-13

**Authors:** Marcos Aviles, José Manuel Alvarez-Alvarado, Jose-Billerman Robles-Ocampo, Perla Yazmín Sevilla-Camacho, Juvenal Rodríguez-Reséndiz

**Affiliations:** 1 Facultad de Ingeniería, Universidad Autónoma de Querétaro, Santiago de Querétaro 76010, Mexico; jmalvarez@uaq.mx; 2 Programa de Postgrado en Energías Renovables, Universidad Politécnica de Chiapas, Suchiapa 29150, Mexico; jrobles@upchiapas.edu.mx (J.-B.R.-O.); psevilla@upchiapas.edu.mx (P.Y.S.-C.); 3 Departamento de Ingeniería Energética, Universidad Politécnica de Chiapas, Suchiapa 29150, Mexico; 4 Departamento de Ingeniería Mecatrónica, Universidad Politécnica de Chiapas, Suchiapa 29150, Mexico

**Keywords:** RNN, GRU, LSTM, bidirectional recurrent neural networks, GWO, metaheuristic algorithms, EMG

## Abstract

Accurate classification of electromyographic (EMG) signals is vital in biomedical applications. This study evaluates different architectures of recurrent neural networks for the classification of EMG signals associated with five movements of the right upper extremity. A Butterworth filter was implemented for signal preprocessing, followed by segmentation into 250 ms windows, with an overlap of 190 ms. The resulting dataset was divided into training, validation, and testing subsets. The Grey Wolf Optimization algorithm was applied to the gated recurrent unit (GRU), long short-term memory (LSTM) architectures, and bidirectional recurrent neural networks. In parallel, a performance comparison with support vector machines (SVMs) was performed. The results obtained in the first experimental phase revealed that all the RNN networks evaluated reached a 100% accuracy, standing above the 93% achieved by the SVM. Regarding classification speed, LSTM ranked as the fastest architecture, recording a time of 0.12 ms, followed by GRU with 0.134 ms. Bidirectional recurrent neural networks showed a response time of 0.2 ms, while SVM had the longest time at 2.7 ms. In the second experimental phase, a slight decrease in the accuracy of the RNN models was observed, standing at 98.46% for LSTM, 96.38% for GRU, and 97.63% for the bidirectional network. The findings of this study highlight the effectiveness and speed of recurrent neural networks in the EMG signal classification task.

## 1. Introduction

The classification and analysis of electromyographic (EMG) signals have emerged as essential research fields in biomechanics and neuroscience. By reflecting the electrical activity produced by the muscles, these signals offer a detailed overview of muscle functionality and potential associated pathologies [1,2]. However, extracting meaningful information from these signals for practical applications requires advanced and efficient processing techniques. Traditionally, EMG analysis has relied on feature extraction to interpret the information, that could help improve the quality of life of people, in different applications [2,3]. However, recurrent neural networks (RNNs) have opened doors to new analysis methods [4].

Using the segmented signal directly instead of extracting features to feed into the network offers a series of advantages that are crucial to the efficiency and quality of the analysis [4,5]. Firstly, working with the totality of the information in the signal allows the preservation of details that, although they may be subtle, are essential. Feature extraction often involves dimensional reduction that could omit important aspects of the signal. Furthermore, this direct approach minimizes complexity in the preprocessing stage by avoiding a meticulous process based on specific domain knowledge [6]. While a set of selected features might be adequate for one application, it may be insufficient for another. In that sense, the segmented signal-based model becomes more flexible. Likewise, neural networks, particularly RNNs, have a remarkable ability to detect complex patterns in data. Feeding the network with the raw signal allows it to identify and learn patterns that may not be evident in a manual extraction process [7].

Despite the promising benefits they present, RNNs are not without significant challenges. One of the main obstacles is the proper selection of hyperparameters, which significantly influence network performance [8]. The adequate selection of hyperparameters in deep learning models is a critical task, but at the same time, it is highly challenging. Hyperparameters, unlike parameters, are not learned during training but are set beforehand. An incorrect choice leads to problems such as overfitting, where the model performs exceptionally well on the training data but fails when faced with unseen data, or underfitting, where the model fails to capture the underlying complexity of the data [9]. Manually searching for these values is notoriously laborious and dependent on the knowledge of the researcher. Although there are automatic techniques, such as grid search or random search, these are computationally expensive [10]. In this context, the Grey Wolf Optimization (GWO) algorithm emerges as a promising solution for hyperparameter selection. This is a metaheuristic algorithm inspired by the social and hunting behavior of gray wolves. The advantages of using GWO lie in its ability to explore and exploit the hyperparameter search space simultaneously [11] and its fast convergence compared with alternative optimization algorithms [12].

Based on the above, this work proposes that through using a combination of an RNN and GWO to analyze EMG signals, an accurate classification is achieved, and traditional limitations in the analysis of these signals are overcome. Long short-term memories (LSTMs), gated recurrent units (GRUs), and bidirectional recurrent neural networks, each with their unique characteristics, are used to capture the complex and sequential nature of EMG signals. LSTMs are notable for their ability to learn long-term dependencies, which is crucial given that EMG signals can contain classification-relevant information over long periods. GRUs, on the other hand, offer an efficient and less computationally intensive option, ideal for real-time applications where resources may be limited. Additionally, bidirectional recurrent networks provide a more complete view of the data by processing information in both directions, ensuring that the context of the entire sequence is taken into account for more accurate classification [13,14,15]. By integrating these advanced RNN methods with the GWO technique, the ability of the model to identify patterns in the data is further improved, resulting in superior performance and greater accuracy in classifying movements based on EMG signals. The contributions of this work are the following:

- Implement a novel methodology using the GWO algorithm to extract features from EMG signals using recurrent neural networks, thereby improving accuracy.
- This approach compares three RNN structures by establishing a solid baseline. This provides a rigorous foundation for evaluating the improvements that each structure can bring to the system performance by integrating a GWO algorithm.

The document is structured as follows. Section 2 reviews previous research addressing issues similar to those discussed here. Section 3 reviews the theoretical foundation, providing a vision of the techniques used. Later, in Section 4, the implemented methodology is detailed. The experiments and their respective results are detailed in Section 5, while Section 6 focuses on an in-depth analysis of the findings. Finally, the study is concluded in Section 7.

## 2. Related Works

Xie et al. [16] developed an advanced neural network model, Bi-ConvGRU, to recognize hand gestures from EMG signals, allowing detailed measurement of muscle activity. This model was evaluated by considering 18 hand gestures from the Ninapro dataset performed by both amputee and non-amputee individuals. The results highlight the potential of this approach for a bio-intuitive and non-invasive control of upper limb prostheses with a physiologically acceptable latency. In [17], the researchers developed a gesture classifier using an RNN model with LSTM layers specifically for hand control in prosthetics. A notable contribution of the authors was enhancing the model’s adaptability for embedded systems by reducing the number of EMG channels.

Metaheuristic algorithms are already used in the field of machine learning. Ref. [18] introduced variations in the Artificial Bee Colony algorithm, which they applied in a KNN classification system to discern hand movements. Likewise, they highlight that this proposal can have applications in physical activities and physiotherapy therapies thanks to its notable performance. In [19], an LSTM model was used to classify the gestures from the forearm muscles. The authors compared the proposed neural network against GRU and demonstrated great performance during online classification. The work of Xiong et al. [20] compared techniques based on machine learning and four RNN configurations, such as GRU, LSTM, and the bidirectional alternative of these two. The models were run to classify eight different gestures from a dataset. The results showed that the bidirectional LSTM configuration obtained the best performance compared to the other RNN configurations and the machine learning models. Aviles et al. [10] developed an SVM classifier incorporating genetic algorithms for feature extraction. Two sets of data were used: the first referring to the right upper extremity and the second composed of movements of the right lower extremity. Likewise, Particle Swarm Optimization (PSO) was implemented to compare both algorithms. The SVM-GA approach significantly improves classification, efficiency, and provides a reduction in the number of parameters compared to the PSO-based approach.

To classify flexion, extension, and ramp walking movements, the authors of [21] employed an LSTM due to its strong suitability for processing nonlinear time-series data. Additionally, to enhance accuracy of the model, they integrated a PSO algorithm for fine-tuning the parameters of LSTM. The PSO-LSTM model significantly improved performance compared to the randomly initialized traditional LSTM. Li et al. [6] employed a methodology based on CNN for classification tasks and RNN for handling timing issues. This approach excelled in real-time recognition, accurately classifying 20 distinct hand movement patterns. A hybrid approach for classifying EMG signals was implemented by [22], utilizing a CNN-LSTM model integrated with a kernel-based PCA technique. The findings demonstrate that the PCA-CNN-LSTM method effectively recognizes lower limb activities from the signals. The overview of the related works is presented in Table 1. However, there is a need for continued research and development to create even more effective algorithms that improve the classical models in this field.

## 3. Materials and Methods

This section discusses and analyzes the main concepts of the theoretical foundation and the materials used to develop this work. The development of the RNN and GWO was carried out in Python using TensorFlow. On the other hand, the filtering and segmentation of the EMG signals was carried out in MATLAB 2018b. The equipment used was a laptop with a 12th-generation i7 processor with an RTX 3060 GPU.

### 3.1. Database

The database presented in [10] was used for this study, which is focused on the muscles specified in Table 2. In the analog filtering phase, a combination of a low-pass filter and a Butterworth high-pass filter was used, both implemented with a second-order Sallen–Key topology and with cut-off frequencies of 600 Hz and 10 Hz, respectively. Additionally, a second-order Bainter notch filter was applied to eliminate 60 Hz interference caused by the power supply. Digitization of the signals was performed using a USB-6002 data acquisition device (DAQ).

The study population consisted of 9 participants, aged between 23 and 27 years: five men and four women. All participants were free of pathologies related to the locomotor system and nervous system and did not have amputation conditions or obesity problems. Five different types of arm and hand movements were recorded, including flexion and extension of the arm at the elbow joint, flexion and extension of the fingers, and a resting state. For this purpose, four bipolar channels placed directly over the muscles of interest were used, which are shown in Table 2. Additionally, a reference electrode was placed on the wrist. Each movement was performed for 6 s, followed by a 2 s relaxation period, and was repeated 20 times using a sampling rate of 1.5 kHz.

The Surface Electromyography for the Non-Invasive Assessment of Muscles (SENIAM) recommendations were followed. The SENIAM project is a European initiative focused on superficial electromyography. It seeks to standardize aspects such as electrode placement and signal processing for EMG. SENIAM recommends locating sensors in 30 individual muscles to obtain quality and stable EMG signals. The recommendations include details on the location, orientation, and distance between electrodes, as well as advice for fixation on the skin and the location of the reference electrode [23].

For the placement of the electrodes, a separation of 20 mm between them was ensured, and attention was paid to the specific characteristics of each muscle. Initially, the reference electrode was placed on the dorsal area of the wrist of the right hand. Subsequently, the reading electrodes were placed on the selected muscles, connecting them to the conditioning and acquisition equipment to begin data capture. During acquisition, participants were asked to perform the indicated movements, ensuring rest periods between each repetition and after each type of movement to avoid muscle fatigue.

### 3.2. Recurrent Neural Network

RNNs are a category of neural networks explicitly designed to work with data sequences, especially useful in natural language processing tasks and time-series analysis [24]. This study explored three types of RNNs: LSTM, GRU, and bidirectional recurrent neural networks.

LSTM is a variant of an RNN designed to address the problem of gradient disappearance, a challenge that occurs in traditional RNNs when processing long data sequences. This is achieved through a cell structure containing entry, exit, and forget gates, allowing the network to have long- and short-term memory. LSTMs learn and remember over long sequences and are, therefore, less sensitive to gaps in data sequences [13]. A GRU is another variant of an RNN that, like LSTM, seeks to solve the problem of gradient disappearance. However, unlike LSTM, GRU simplifies the cell structure by merging the input and forget gates into a single update gate. This reduces the computational complexity and, in specific contexts, offers performance comparable to or even superior to LSTM with a shorter training time [14]. Bidirectional neural networks take advantage of sequence information in both directions (past and future) to improve accuracy in classification and prediction tasks. This is achieved by running two traditional RNNs: one that moves forward through the sequence and one that moves backward. Both outputs combine to provide a more informed perspective on the sequence, which can result in better accuracy on specific tasks [15]. Algorithm 1 shows the programming logic to implement RNNs with GWO.
**Algorithm 1** Optimization of bidirectional neural network, LSTM, and GRU with GWO.  1: **Inputs:** Training data, validation data
  2: **Initial hyperparameters:**
  3: Learning rate: lr∈[0.00001, 0.1]
  4: Neurons in layer 1: $n_{1}\in \left[10,150\right]$
  5: Neurons in layer 2: $n_{2}\in \left[10,150\right]$
  6: Batch size: batch_size∈[128,512]
  7: Training epochs: epochs∈[10,100]
  8: **procedure** CreateModel($lr,n_{1},n_{2}$)
  9:        Initialize RNN model with defined structure10:        Add RRN layer units of $n_{1}$ neurons11:        Add RRN layer units of $n_{2}$ neurons12:        Add dense layer for classification13:        Compile model with learning rate lr14:        **return** model15: **end procedure**16: **procedure** FitnessFunction(hyperparameters)17:        $lr,\;n_{1},\;n_{2},\;batch\_size,\;epochs\leftarrow hyperparameters$18:        model←CreateModel($lr,n_{1},n_{2}$)19:        Train model with epochs and batch_size20:        Evaluate model on validation data21:         **return** validation error22: **end procedure**23: **Optimize with GWO:**24: Define search space with defined ranges25: optimal_hyperparameters← GWO(FitnessFunction)26: $optimal\_lr,\;optimal\_n_{1},\;optimal\_n_{2},\;optimal\_batch\_size,\;optimal\_epochs\;\;\leftarrow$      optimal_hyperparameters27: **Train and validate with selected hyperparameters:**28: optimal_model←CreateModel($optimal\_lr,optimal\_n_{1},optimal\_n_{2}$)29: Train optimal_model with optimal_epochs and optimal_batch_size

### 3.3. Hyperparameters

A hyperparameter is a parameter not intrinsically derived from the data but set before training the model. Hyperparameters guide how the neural network learns and how the model optimizes. Ensuring the appropriate selection of these hyperparameters is essential to achieving exceptional model performance [25].

When working with neural networks such as GRU, LSTM, and bidirectional neural networks, various hyperparameters are essential and drastically influence the behavior of the model. These hyperparameters cover aspects such as the number of units or neurons in the layers, the activation function used, the learning rate, and the total number of training epochs. For example, the number of units in the layers largely determines the ability of the network to model complex interactions in the data. Increasing this number can allow the network to understand more sophisticated patterns but also runs the risk of overfitting [26].

The activation function introduces nonlinearity into the network, thus its ability to model nonlinear relationships. Although the sigmoid function is recognized, in networks such as LSTM or GRU, functions such as the scaled exponential linear unit (SELU) are frequently used [27]. Regarding the learning rate, this regulates the magnitude of adjustment of the weights in each training cycle. Too high a rate can cause oscillations in the network, preventing convergence, while an excessively low rate can cause slow convergence, trapping the model in local minima. On the other hand, the number of epochs establishes how often the entire dataset is used during training, which is crucial to avoid overfitting or underlearning [8].

Table 3 shows the hyperparameters adjusted using GWO to determine the most suitable values in the GRU, LSTM, and bidirectional recurrent neural networks.

### 3.4. Grey Wolf Optimizer

GWO is a metaheuristic optimization algorithm proposed by [11], inspired by the social and hunting behavior of gray wolves. Its design emulates the hierarchical structure and hunting tactics these creatures deploy in nature. Hierarchy in gray wolves: Gray wolves have a very marked hierarchical social structure in the wild. Within this hierarchy, four main types of wolves stand out:

- Alpha (α): They are the leaders of the pack, usually a couple (male and female). They make all the critical decisions, from the time of hunting to the time of migrating or resting.
- Beta (β): They are second in command. If both alphas die, the beta would assume leadership. They help the alphas make decisions and act as an “advisor”.
- Delta (δ): They act as guardians of the pack. They protect the wolves from external threats and maintain order within the group.

The GWO uses this hierarchy to update the wolves’ (solutions) positions in the search space. The rankings are updated based on the position of the top three wolves (α, β, δ). The position of the rest of the wolves is updated based on these three best positions, emulating hunting and tracking behavior. The hunting process is modeled mathematically using equations that represent the pursuit, encirclement, and attack of prey. These equations are based on the distance between the wolf and its prey and are adjusted according to the hierarchy. One of the main advantages of GWO is its balance between exploration (looking for new areas in the solution space) and exploitation (refining a solution in a specific area). This is due to hierarchical and cooperative behavior of the wolves when hunting, which allows the algorithm to evade local optima and converge towards a suitable global solution.

Hunting behavior is imitated using hunting coefficients. For each wolf (except alpha), the following coefficients are used [11]:

- $A_{1},A_{2},A_{3}$: These coefficients define the magnitude of the attraction towards the leading wolves. They control the ability of the wolves to explore and exploit.
- $C_{1},C_{2},C_{3}$: These coefficients are random vectors obtained for each iteration and wolf. They help in adjusting the position of each wolf concerning the leading wolves.
  The hunting coefficients are typically calculated as follows:
  (1) $$\begin{array}{cc} A_{ij} & =2a\times r_{1}-a \end{array}$$
  (2) $$\begin{array}{cc} C_{ij} & =2\times r_{2} \end{array}$$
  where $r_{1}$ and $r_{2}$ are random numbers in [0, 1], and *a* decreases linearly from 2 to 0 over the iterations. For each wolf in the group (except the leading wolves), the distances to the three leading wolves are calculated as follows:
  (3) $$\begin{array}{cc} D_{\alpha } & =|C_{1j}\times X_{\alpha }-X_{i}| \end{array}$$
  (4) $$\begin{array}{cc} D_{\beta } & =|C_{2j}\times X_{\beta }-X_{i}| \end{array}$$
  (5) $$\begin{array}{cc} D_{\delta } & =|C_{3j}\times X_{\delta }-X_{i}| \end{array}$$
  where $X_{\alpha },\;X_{\beta }$, and $X_{\delta }$ are the positions of the alpha, beta, and delta wolves, respectively, and $X_{i}$ is the position of the current wolf. These distances are then used to adjust the position of each wolf based on the positions of the leading wolves. The goal is to bring the wolves closer to the best solutions in the search space, guiding the pack toward possible optimal solutions.
  Algorithm 2 shows the programming logic to implement GWO.

**Algorithm 2** Grey Wolf Optimizer.  1: **Inputs:** Objective function f(x), Population size *N*, Maximum iterations *T*  2: **Initialization:**  3: **for** i=1 to *N* **do**  4:        Initialize wolf position $X_{i}$ randomly  5:        Calculate fitness $f(X_{i})$  6: **end for**  7: **for** t=1 to *T* **do**  8:        **Update coefficients:**  9:        $a=2-t\times \frac{2}{T}$10:        **Update alpha, beta, and delta wolves:**11:        Identify the top three wolves $X_{\alpha }$, $X_{\beta }$, $X_{\delta }$ based on fitness12:        **for** i=1 to *N* **do**13:                **for** j=1 to dimension **do**14:                       Calculate hunting coefficients $A_{1},C_{1},A_{2},C_{2},A_{3},C_{3}$15:                       Calculate distances $D_{\alpha },D_{\beta },D_{\delta }$16:                       Update position using $X_{\alpha },X_{\beta },X_{\delta }$17:                **end for**18:                Apply boundary conditions if necessary19:                Update the fitness of $X_{i}$20:        **end for**21: **end for**22: **Output:** Best solution $X_{\alpha }$

### 3.5. Windowing

The windowing technique is widely used in signal processing and time series to divide a continuous dataset into more manageable segments called windows. This technique is advantageous when analyzing data that undergo temporal variations, such as electromyographic or electrocardiographic signals. A common and notable variant of windowing is the use of overlapping windows. Unlike segmentation into discrete, non-overlapping windows, overlapping windows allow an overlap between consecutive windows by a given number of points. Overlapping windows offer several advantages: an improvement in temporal resolution, a reduction in edge error, and an increase in data density. The improvement in temporal resolution is because the overlap between windows allows us to detect events or features in the data that could go unnoticed or not be clearly defined in a segmentation without overlap. Reducing edge error is an essential benefit since, in some applications, the start and end of a window can introduce artifacts or errors. These errors can be minimized by overlapping windows since data at the edges of a window are also analyzed in the context of the adjacent window. Finally, the increase in data density refers to the fact that segmentation with overlapping windows generates a more significant number of segments for the same dataset compared to segmentation without overlap, which can benefit machine learning techniques by providing more examples to train and validate models [28,29].

When implementing overlapping windows, it is crucial to consider the degree of overlap, generally defined as a percentage of the window size. It should be noted that a more significant overlap increases the correlation between consecutive windows, which can be beneficial for detecting subtle transitions in the data. However, it can also introduce redundancy [30].

## 4. Methodology

This section outlines the methodological steps undertaken to implement this work.

### 4.1. Signal Processing

The first step in processing consisted of filtering the signals to attenuate noise. Since the original data sequence of the EMG signal was used, the classification of the signals may have been susceptible to interference and artifacts. Therefore, it was essential to perform filtering before proceeding with window segmentation. For this purpose, a second filtering stage was used in addition to the analog filtering of the database. In this case, it corresponded to a second-order digital Butterworth bandpass filter with cut-off frequencies between 10 and 500 Hz, which were the frequencies of interest, using the “Butter” and “filtfilt” functions of the MATLAB 2018b software [31].

After filtering, the signals were segmented into 250 ms windows, overlapping by 190 ms [28,29]. It is important to note that the EMG signal contains 2 s of rest before the start of the movement. Therefore, these were discarded to focus exclusively on the information generated by the movement of the arm. After removing these 2 s, the remaining signal was divided into 63 windows. The choice of using overlapping windows is due to their ability to continuously collect information during the operation of the classification algorithm, which is essential for its real-time application. Additionally, using this approach increases the cadence of classification decisions since each analysis window requires less data to complete, in this case, 250 ms.

After extracting the windows, the information was organized to be introduced into the neural networks in a three-dimensional matrix of dimensions *i*, *j*, and *k*. Here, *i* represents the total number of windows for each acquisition in the database, calculated as 9 people × 20 trials × 5 movements × 63 windows, resulting in i=56,700. The dimension *j* is related to the number of sensors used in each acquisition, which is four. Meanwhile, *k* represents the total number of data points found in each window, with a total of 375 points per window. This value reflects the data collected in a time interval of 250 ms, with a sampling rate of 1.5 kHz.

The experimentation was developed in two stages. In the first, the applicability of the method to EMG signals was validated, involving most volunteers in the training and validation phases. In contrast, the second stage was designed to evaluate the robustness of the methodology, using a higher percentage of individuals in the testing phase.

In the first stage, the generated matrix was organized so that the data of the first eight people were allocated to the training and validation phases, reserving the information of the ninth individual exclusively for testing. Of the set of 8 people, 80% of their data were used for training and the remaining 20% for validation. It is vital to highlight that these subsets were mixed randomly to prevent any possibility of overfitting in the network.

A second experimental round was carried out to check the efficiency and viability of the proposed method. In this second round, a ratio of 5 to 4 of the people was used. This means that five individuals were used for the training and validation round, while the remaining four were used to testing the models. On this occasion, the 80–20 division for training and validation was also respected.

It is relevant to note that the validation accuracy is used for hyperparameter tuning. This is evident in Algorithm 1, specifically in lines 20 and 21. On the other hand, the testing accuracy is used to confirm the model results. The testing set is made up exclusively of subjects not included in the training and validation sets. It is also important to mention that the models were trained from scratch in both stages. The accuracy calculation is presented in Equation (6). This equation defines accuracy as the ratio of correct predictions to the total number of predictions [10].

(6) $$Accuracy=\frac{TP+TN}{TP+TN+FP+FN}$$

where TP represents true positives, the cases in which the model correctly predicts the positive class. TN refers to true negatives, cases where the model correctly identifies the negative class. FP indicates false positives, which occur when the model incorrectly predicts a positive outcome for a case that is negative. Finally, FN are false negatives, in which the model fails to recognize the positive class, erroneously classifying it as harmful.

Sensitivity, also known as true positive rate, measures the proportion of correctly predicted positive instances to all actual positive instances. It focuses on the ability of the model to capture all positive instances and avoid false negatives. Equation (7) shows the equation that defines the sensitivity.

(7) $$Sensitivity=\frac{TP}{TP+FN}$$

Specificity, or true negative rate, measures the proportion of correctly predicted negative instances to all true negative instances. It indicates the ability of the model to identify negative examples correctly and is crucial for its discriminative power. Equation (8) shows the equation that defines the specificity.

(8) $$Specificity=\frac{TN}{TN+FP}$$

### 4.2. Recurrent Neural Networks

Within the framework of this work, three variants of recurrent neural network architectures were designed and implemented, namely, LSTM, GRU, and a bidirectional neural network. These architectures were implemented using Python, relying on the TensorFlow library. Two recurrent layers are included in each of these architectures, and the SELU activation function is used. The Adam optimizer was selected to adjust the weights, while, to evaluate the performance of the model, the accuracy metric was adopted. The cross-entropy loss function was used during the training phase to calculate the error.

As the output of the model, a dense layer composed of five neurons was added, one for each movement, using the softmax activation function. Furthermore, to stabilize the activations and facilitate training, a normalization layer, specifically LayerNormalization, was included before the recurrent layers.

The early_stopping callback was integrated to optimize training time, which stops training if no improvement in accuracy is perceived in the validation dataset for five consecutive iterations. The number of iterations executed during training served as feedback for the GWO algorithm, allowing the number of epochs needed to achieve the best results to be adjusted.

Bidirectional networks can be built using GRU or LSTM structures as a base. In our particular case, LSTM layers were used for the bidirectional configuration. Additionally, the computational efficiency of the different architectures was assessed, identifying advantages and disadvantages in terms of training time, memory use, and precision.

### 4.3. Grey Wolf Optimizer

GWO algorithm was implemented in Python, using the numpy and pandas libraries. A population of 20 wolves was established for this optimization, and the algorithm iterated over ten cycles. Table 4 shows the ranges of the hyperparameters to optimize.

At the end of the ten iterations of the GWO, various data of interest were recorded for the best solutions found. These included the best position (representing the suggested hyperparameters), the associated cost (indicating the validation classification error in the neural network), the structure of the obtained neural model, and the corresponding training and prediction times. This process allowed us to fine-tune the configuration of the neural networks, searching for the best combinations of hyperparameters that would minimize the classification error while optimizing the performance and efficiency of the model.

## 5. Results

This section details the results obtained for the two experimental stages described in Section 4.

### 5.1. First Stage

Table 5 shows the hyperparameters achieved for each of the recurrent networks optimized using GWO.

A notable robustness is observed in the LSTM model, with 102 neurons distributed between two layers. It has a total of 76,861 trainable parameters. On the other hand, bidirectional networks have only 27 neurons in total but have 48,183 trainable parameters. On the other hand, the GRU is presented with only 43 neurons and 33,425 trainable parameters. The lighter nature of GRU may be the reason why it takes more epochs to reach convergence. Despite this difference in the density of neurons between GRU and bidirectional networks, there is no considerable disparity in complexity. This observation shows that a more significant number of neurons does not necessarily result in an intrinsically more complex network. Concerning learning rates, a high rate such as the one adopted by the bidirectional model (0.0117) suggests a faster adaptation of the weights, although with possible oscillations that may be experienced during the process. Meanwhile, more contained rates, such as those adopted by the LSTM (0.00346) and GRU (0.00554), suggest a more cautious approach toward convergence.

**Figure 1:**
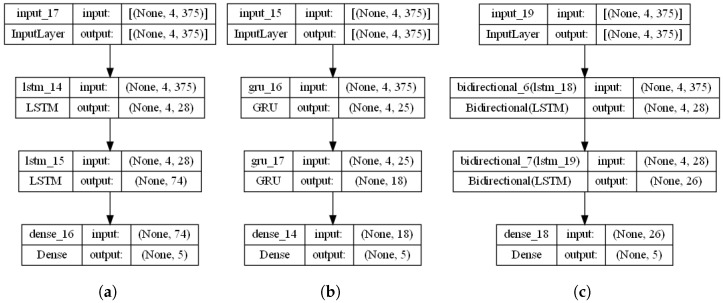
Final block diagram for the three trained and adjusted models, (**a**) LSTM network, (**b**) GRU, and (**c**) bidirectional, for first stage.

The batch size, which is another crucial hyperparameter, shows variations between architectures. In GRU, a considerable batch of 329 is used, probably to speed up training through simultaneous data processing. However, this benefit may be risky, as larger batch sizes may compromise convergence accuracy. Despite these risks, on all architectures, including LSTM with a batch size of 188 and bidirectional with 199, a flawless accuracy of 100% was achieved during testing. Figure 1 shows the final block diagram for each of the three trained models.

Table 6 shows the temporal analysis of the different architectures of the recurrent neural networks studied. A difference in time is observed between the different stages evaluated.

The LSTM network proved to be the most efficient in terms of training time, requiring only 31.47 s. This result is particularly interesting given its high neuronal density and relatively large number of trainable parameters. The moderate learning rate (0.00346) and batch size (188) could contribute to this rapid convergence and efficient training. Regarding validation time, the LSTM was also slightly faster than the GRU, needing only 0.81 s. LSTM was remarkably effective for prediction, with a time of only 0.12 ms.

On the other hand, the GRU, despite being less dense and having fewer parameters than the LSTM, required a longer training time of 51.28 s. Given its lighter architecture, this longer duration is related to the need for more epochs to converge. The validation time of the GRU was slightly longer than that of the LSTM, registering 0.85 s. Despite this marginal difference, it is relevant to mention that the GRU’s prediction time, while still relatively fast, was slower than the LSTM, taking 0.134 ms.

Finally, the bidirectional architecture, which uses an underlying LSTM structure to process sequences in both directions, showed the longest training time of the three, at 81.60 s. This increase in time is associated with the bidirectional nature of the model, which processes forward and backward information, intrinsically increasing the computational load. Despite its compact configuration of neurons, its validation time was the longest, requiring 1.23 s. In terms of prediction, it also showed the longest time, at 0.2 ms.

**Figure 2:**
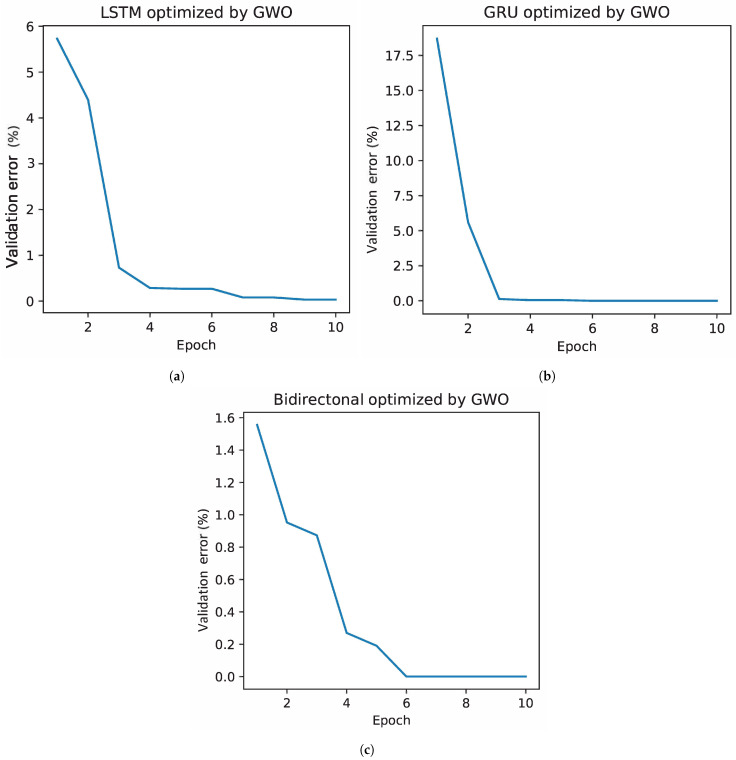
Reduction in the error classification due to the selection of hyperparameters by GWO. Where (**a**) represents the error in the LSTM, (**b**) in the GRU, and (**c**) in the bidirectional network.

Figure 2 presents the error evolution in different recurrent networks through the GWO optimization method. The GRU network, shown in Figure 2b, starts with the highest error, approximately 17.5%. However, its rapid convergence is notable, reaching an error of 0% in the third iteration. On the contrary, the bidirectional network, which can be seen in Figure 2c, starts with the lowest error, 1.6%, in its first iteration, thanks to an appropriate combination of hyperparameters obtained by the algorithm. Despite this, it requires six iterations to minimize the error to 0%, showing a more gradual reduction than the other architectures, a direct consequence of its low starting error. In the case of the LSTM, presented in Figure 2a, it starts with an error of 6% and shows a rapid decrease until the third iteration, after which its decrease becomes more gradual, reaching 0% in the eighth iteration.

**Figure 3:**
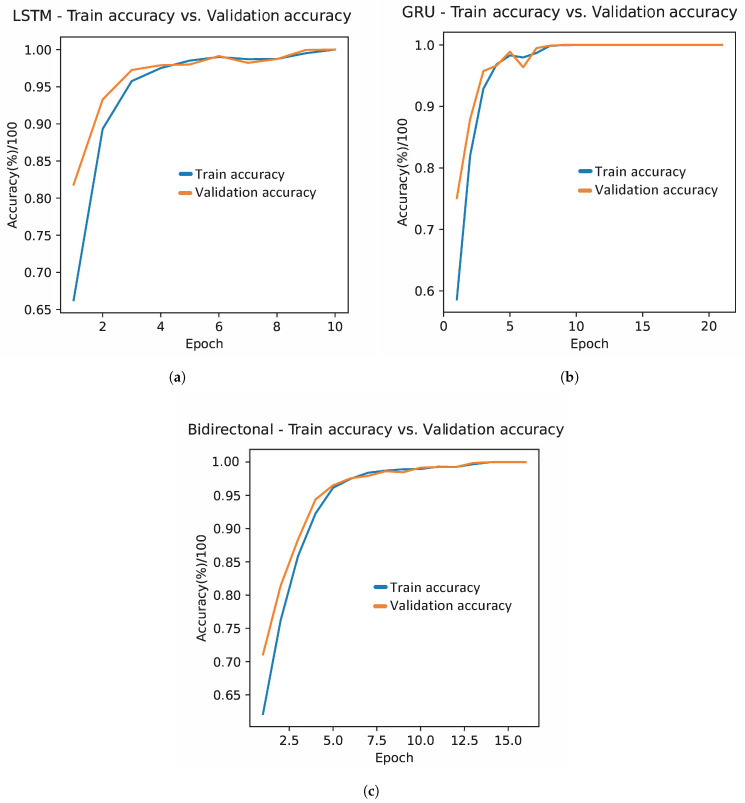
Evolution of training and validation accuracy with hyperparameters defined by GWO in the first stage. Where (**a**) represents the accuracy evolution in the LSTM, (**b**) in the GRU, and (**c**) in the bidirectional network.

Figure 3 illustrates the training and validation accuracy behavior of the three recurrent neural network models: LSTM, GRU, and bidirectional, each optimized with the GWO optimization algorithm. Consistently across all three models, an increase in classification across iterations is observed, indicative of an absence of overfitting. The LSTM model shows a rapid increase in accuracy that soon stabilizes, maintaining a slight advantage in training accuracy over validation, suggesting effective generalization without falling into memorization. On the other hand, although the GRU model follows a similar trend in increasing precision, it presents a distinctive peak in the validation curve that could be attributed to temporal overfitting or variations in the test data. However, this model also stabilizes its precision, demonstrating its ability to adapt and generalize with the advancement of time. The bidirectional network maintains the general behavior observed in LSTM and GRU, with the training and validation accuracy curves advancing in close formation throughout the process.

**Figure 4:**
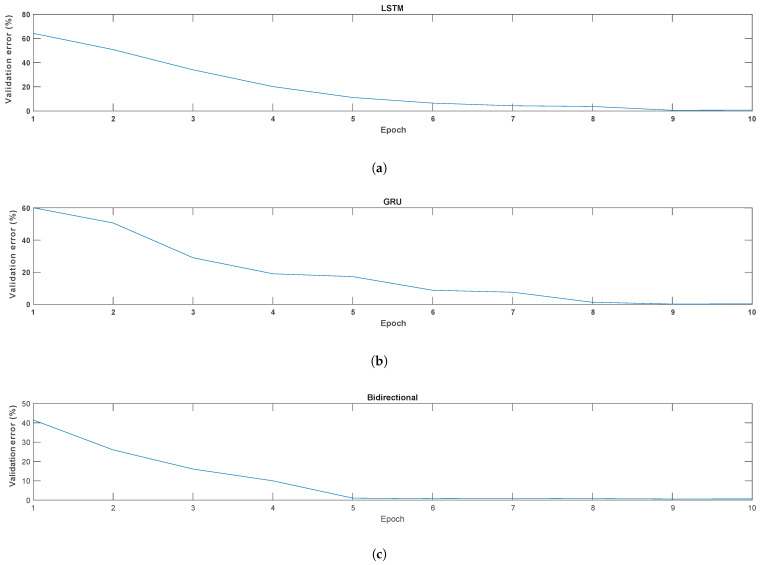
Evolution of mean validation error for LSTM, GRU, and bidirectional recurrent neural networks. Where (**a**) represents the mean validation error in the LSTM, (**b**) in the GRU, and (**c**) in the bidirectional network.

Figure 4 presents the evolution of the average error in the wolf population throughout the iterations, illustrating how the global solutions improve as they advance. A distinctive feature of metaheuristic algorithms is their ability to offer multiple solutions at the end of the iterative process. Each solution, corresponding to an individual in the population, can be adapted to the desired objective but with different properties. At the end of iteration 10, several RNN configurations reported an error of less than 1%, each with different sets of hyperparameters. For this study, those networks with faster response times in the evaluation stage of each topology were chosen. However, it is possible to select networks according to other criteria, such as the minimum number of neurons or the shortest training time, depending on the specifics of the problem addressed.

Figure 4a, corresponding to LSTM, reveals a start with the highest average error, approximately 65%. Furthermore, it shows a convergence to the lowest error in iteration 9, characterized by a gradual decrease. This behavior suggests a constant and balanced optimization of the prediction for the LSTM population. In contrast, Figure 4b, corresponding to GRU, exhibits a more irregular evolution, with an initial error close to 60%, reaching the minimum average error at iteration 8. This slightly oscillating behavior in GRU suggests that the GWO algorithm faces challenges in finding solutions that significantly reduce the error. Finally, Figure 4c shows that bidirectional neural networks start with a lower average error, around 41%. These networks reach faster convergence, achieving the minimum error in iteration 5. Their smooth and rapid trajectory suggests that GWO has a better facility to identify favorable solutions in this topology.

This study used SVM with a Gaussian kernel as a reference model. Since SVM does not allow direct processing of raw signals, performing a proper characterization of these signals was imperative. The characteristics proposed in ref. [10] were used for this. The features used are shown in Table 7.

It is relevant to highlight that the dataset and features used in this study are the same as those used in [10]. These features were carefully selected for this database in the previously mentioned work. By implementing the above-mentioned features, the SVM model achieved an efficiency of 93%. Table 8 presents the fundamental comparisons between the models based on RNN and SVM.

Table 9 provides a detailed analysis of the performance of an SVM classifier in the testing stage for the five moves. Regarding sensitivity, class 1 shows the best performance with 85.2%, closely followed by class 2 with 81.9%. Class 3 also performs well, with 80.2%. However, the sensitivity decreases noticeably for classes 4 and 5, with 63.9% and 72.1%, respectively, indicating that the SVM classifier has difficulty correctly identifying these classes compared to the first three. Regarding specificity, which evaluates the classifier’s ability to correctly identify negatives, a generally high performance is observed in all classes. Class 1 achieves a specificity of 95.6%, and classes 2 and 3 also exhibit high specificity, 93.2% and 95.9%, respectively. Although classes 4 and 5 present lower specificity, 83.2% and 82.9%, these values are still relatively high. It is important to contrast these results with the performance achieved by the LSTM, GRU, and bidirectional models which, by achieving 100% accuracy, also achieve 100% sensitivity and specificity. The lower performance of the SVM, particularly in sensitivity for classes 4 and 5, could indicate limitations in its ability to handle certain characteristics of these data or require more specific tuning of the model.

Table 8 shows an interesting comparison concerning the training and response times of the models. Noteworthy is the fact that SVM has the shortest training time. However, this efficiency is offset by a longer response time in the classification phase. This behavior is attributed to extracting features from the data before entering them into the classifier. This additional step imposes a delay that affects its performance in terms of response time. In contrast, RNN networks have the advantage of working directly with the raw data, eliminating the need for a feature extraction step and offering faster response.

Another relevant aspect is classification efficiency. Even though all models are trained using the same database and identical preprocessing, SVM has a lower classification rate. This discrepancy is due to the added complexity of selecting appropriate features. While the effectiveness of RNNs focuses on the quality and complexity of the input data, SVM has the particularity of depending not only on the selected features but also on the interaction and synergy between them. This analysis highlights the fundamental differences between feature-based approaches and those based on raw data, highlighting the strengths and limitations inherent to each methodology in the context of EMG signal classification.

### 5.2. Second Stage

Table 10 shows the hyperparameters achieved for each of the recurrent networks optimized using GWO in the second stage.

For the LSTM model, an increase in the number of neurons in the first layer is observed from 28 to 31, suggesting a need for greater capacity to adapt to the variability in the data in the second stage, where a more significant number of individuals was included in the testing set. However, there was a significant reduction in the number of neurons in the second layer, going from 74 to 13, which could indicate an attempt to simplify the model to prevent overfitting. The batch size increased from 188 to 206, while the training epochs increased from 10 to 14, indicating that the model required more iterations on the data to reach convergence. Additionally, there was a slight increase in the learning rate.

In this second stage, a significant change is observed in the configuration of the GRU model. The number of neurons in the first layer was slightly reduced to 22, while it was increased to 101 in the second layer. This redistribution in model capacity suggests a change in modeling strategy, possibly due to differences in variability. By having a more significant number of individuals in the testing set in the second stage, the model may have needed to strengthen the internal layers to better generalize over the unseen data, thus avoiding overfitting the peculiarities of the training set. The batch size experienced a slight increase to 339, and the training epochs decreased to 19. These changes in the training hyperparameters suggest a search for balance between the stability and the speed of convergence of the model. A larger batch size may contribute to a more stable gradient estimation during training. At the same time, the reduction in the number of epochs suggests that the model was able to achieve an excellent fit to the data more efficiently. Finally, the learning rate increased from 0.00554 to 0.00731, indicating a more aggressive adjustment to the model weights during training. This increase can be interpreted as an attempt to speed up the training process.

**Figure 5:**
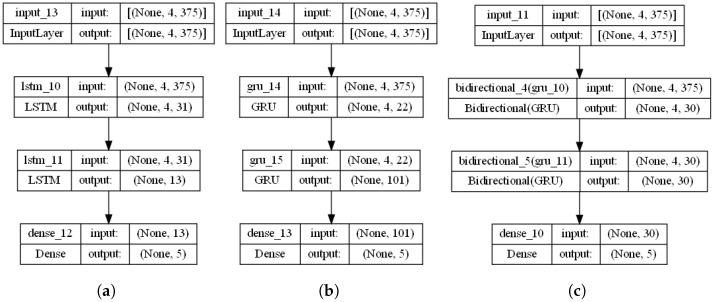
Final block diagram for the three trained and adjusted models, (**a**) LSTM network, (**b**) GRU, and (**c**) bidirectional, for second stage.

In the second stage, the number of neurons in both layers experienced a slight increase, reaching 15 for both for the bidirectional model. This change suggests an adjustment of the model in response to the increased variability in the data introduced by the change in the distribution of the training, validation, and testing sets. It is important to note that a relatively simple structure is maintained despite this increase in the capacity of the model. The batch size was kept constant at 199, indicating that the data processed in each training iteration was adequate from the first stage. However, the training epochs decreased slightly to 15, suggesting that the model was able to fit the data more efficiently in the second stage despite potential additional complexities. One of the most notable changes was the learning rate, which increased from 0.0117 to 0.0177. This increase in the speed at which the model adjusts its weights is an effort to speed up the training process and achieve faster convergence. Figure 5 shows the final block diagram for each of the three trained models.

Table 11 shows the temporal analysis of the different architectures of recurrent neural networks studied in stage two.

In evaluating the training, validation, and prediction times of the different recurrent neural network architectures, distinctive patterns and significant changes are observed between the two stages of the study. The LSTM model proved the most time efficient, with 31.47 s for training, 0.81 s for validation, and 0.12 ms for predictions. However, in the second stage, these times increased, recording 52.76 s, 1 s, and 0.21 ms, respectively. This increase can be attributed to increased training epochs, which implies a higher computational cost.

On the other hand, despite being generally slower than the LSTM, the GRU model maintained reasonable times and experienced a less pronounced increase between the two stages. In the first stage, the GRU recorded 51.28 s, 0.85 s, and 0.134 ms for training, validation, and prediction, respectively, and in the second stage, these times increased to 57.90 s, 1.2 s, and 0.24 ms. This behavior may be related to the adjustments to the number of neurons and the learning rate observed in the hyperparameters.

The bidirectional neural network, for its part, showed the highest times in both stages, underlining its computationally more intensive nature due to information processing in two directions. In the first stage, the times were 81.60 s for training, 1.23 s for validation, and 0.2 ms for predictions, while in the second stage, these increased dramatically to 115.16 s, 2.6 s, and 0.34 ms, respectively. This increase in times can be justified by the increase in the complexity of the model, reflected in the number of neurons and the learning rate.

**Figure 6:**
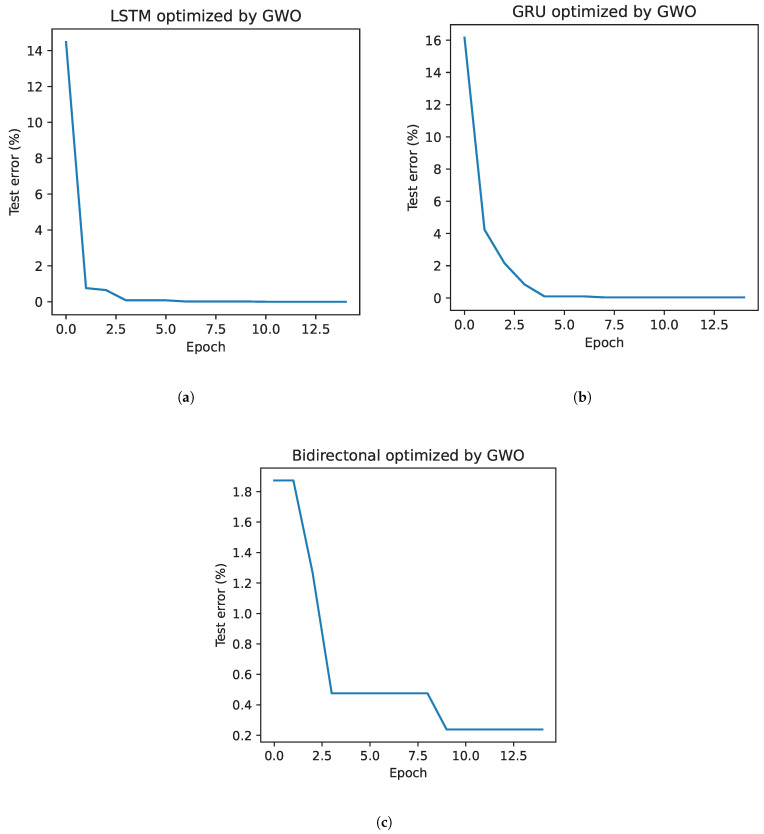
Reduction in the error due to the selection of hyperparameters by GWO in the second stage. Where (**a**) represents the error in the LSTM, (**b**) in the GRU, and (**c**) in the bidirectional network.

Figure 6 presents the evolution of the best solution per iteration of the GWO optimization algorithm applied to the data from the second stage. In this instance, particular behaviors can be observed in each of the neural network architectures evaluated. In the case of the LSTM network, Figure 6a, it starts with an error close to 14%, which is higher than that recorded in the first stage. However, this network shows a remarkable ability to quickly adjust its parameters, resulting in an accelerated decrease in error. This phenomenon can be attributed to the reduction in the number of individuals used in the training and validation phases, decreasing the variability in these datasets and facilitating the network learning process. On the GRU network side, shown in Figure 6b, a similar initial behavior is observed in both stages, with a comparable starting error. However, during the second stage, the decrease in error manifests itself more gradually, reaching a minimum in the fourth iteration for both phases of the experiment. Finally, in Figure 6c, the bidirectional network presents a less abrupt error decay during the second stage, reaching a minimum error in iteration 9. This contrasts with the first phase, where the minimum error is achieved in iteration 6.

**Figure 7:**
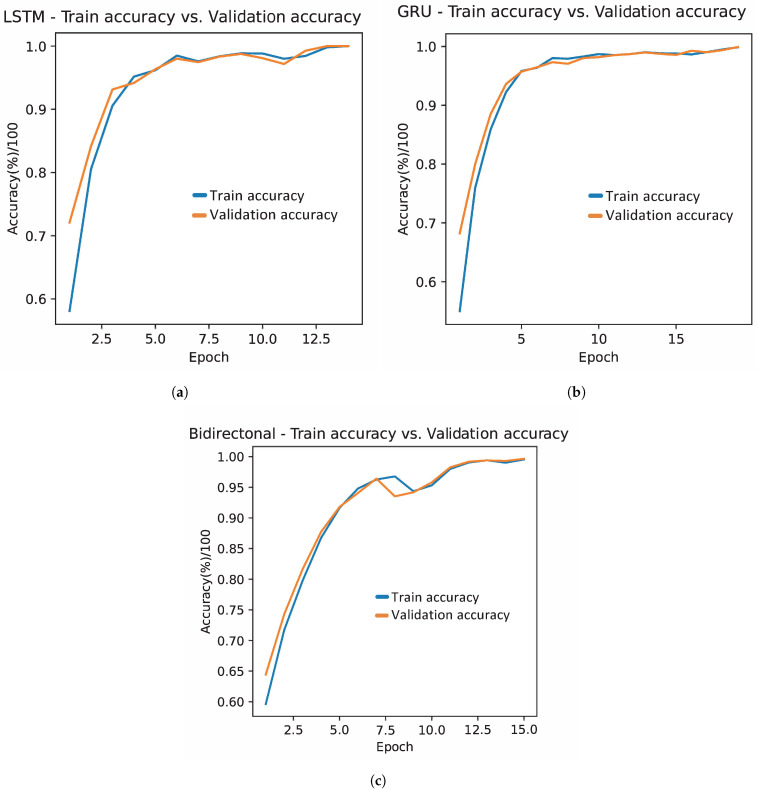
Evolution of training and validation accuracy with hyperparameters defined by GWO. Where (**a**) represents the evolution of the accuracy in the LSTM network, (**b**) in the GRU network, and (**c**) in the bidirectional network.

Figure 7 illustrates an encouraging behavior of the models during the training and validation phases, highlighting the absence of overfitting, since a concurrent increase in precision is observed for both phases. However, it is particularly interesting to note the peculiar behavior of the bidirectional network between iterations 8 and 11, where a brief decrease in percentage accuracy is experienced, as shown in Figure 7c. This small valley in accuracy could be attributed to a slightly high learning rate, which could have caused oscillations in model convergence. However, the crucial thing to highlight is the ability of the bidirectional network to recover, eventually achieving a classification close to 99%. This demonstrates the notable resilience and robustness of the model, highlighting its ability to overcome temporary setbacks in training and improve its accuracy.

Table 12 summarizes the precision achieved by each model in the testing phase for each experimental stage. During the first stage, the LSTM, GRU, and bidirectional models achieved an impressive 100% accuracy, highlighting their ability to capture and learn from the complexity of arm movement patterns based on EMG signals. However, in the transition to the second stage, a slight decrease in the accuracy of all models was observed. The LSTM model, initially achieving classification perfection, experienced a slight drop, recording 98.46% accuracy. This decrease is attributed to the variability introduced by the new distribution of individuals in the training and testing phases. For its part, despite having maintained an accuracy of 100% in the first stage, the GRU model showed a more pronounced decrease in the second, reaching 96.38% accuracy. This reduction is due to its simpler structure compared to the LSTM, making it more susceptible to variations in the data. Despite its ability to process information in both directions and capture more complex contexts, the bidirectional network was not immune to variability between stages and experienced a decrease in accuracy, registering 97.63% in the second stage. Although this decrease is notable, the bidirectional network managed to maintain relatively high performance, demonstrating its robustness and ability to adapt.

The results presented in Table 13 reveal the performance of the three implemented models regarding sensitivity and specificity across five different classes. In general, all models exhibit high sensitivity and specificity in all classes, with most values exceeding 95%. This demonstrates a strong ability of the models to identify instances of each class (sensitivity) correctly and to properly exclude instances that do not belong to that class (specificity). Furthermore, there is notable consistency in performance across different classes for each model, suggesting good generalization of the models across various classification conditions.

Analyzing each model individually, LSTM achieves the highest sensitivity and specificity rates in almost all classes for values greater than 97%. On the other hand, although achieving a sensitivity and specificity of 100% in classes 1 and 3, respectively, the GRU model shows slightly lower performance in other classes compared to the LSTM, being more notable in classes 4 and 5, where the sensitivity drops below 95%. The bidirectional model shows behaviors similar to LSTM and GRU. Regarding the analysis by class, classes 1 to 4 are those that the three models most accurately identify. However, class 5 is the most challenging regarding sensitivity, especially for the GRU model. This could suggest greater complexity or similarity to other classes that make their precise identification difficult.

## 6. Discussion

This study conducted a meticulous comparative analysis between various recurrent neural network architectures, including LSTM, GRU, and bidirectional, evaluating crucial aspects such as accuracy, training times, and testing and prediction capabilities. Using the GWO optimization method to tune the hyperparameters, exceptional accuracy was achieved during the evaluation stage, reaching 100% on all RNN models during the first experimental phase. These results highlight the effectiveness of RNNs in processing EMG data with minimal preprocessing. However, when advancing to the second experimental phase, a decrease in precision was observed, obtaining 98.46% for LSTM, 96.38% for GRU, and 97.63% for bidirectional. Despite this level of reduction, the RNN models continued to demonstrate outstanding performance, underscoring their robustness and reliability in the classification task. Likewise, a nonlinear relationship is observed between the number of neurons and the computational complexity of the networks. Although intuitively this can be interpreted as, the greater the number of neurons, the greater the complexity, this study revealed that this is not always the case. Despite having the smallest number of neurons, bidirectional recurrent neural networks proved to be as complex as LSTM and more complex than GRU in terms of trainable parameters.

In this study, training, validation, and prediction times varied significantly between recurrent neural network architectures. The LSTM model stands out for its temporal efficiency, indicating more agile processing. On the other hand, the GRU and bidirectional models show longer times, which suggests a greater demand on processing resources, possibly due to more elaborate structures and adjustments in their hyperparameters. These differences reflect how each architecture handles tasks, providing insight into their operation and efficiency in different scenarios.

It is worth highlighting, however, some limitations of the present study. Although an extensive set of 56,700 data windows was available, these come from only nine individuals, raising questions about the generalization capacity of the models. This aspect highlights the need to increase the dataset by including information from a more diverse group of participants to strengthen the validity of the inferences made. Regarding the sensor configuration, only four sensors were used to differentiate five different movements. Based on previous work [10], this opens a field for future research, exploring how RNNs could behave under an even greater variety of movements with a reduced set of sensors. The discrimination capacity of RNNs in these circumstances constitutes a promising and highly relevant line of research.

Finally, expanding the database and considering the multiple solutions GWO can offer is essential. Although they converge towards a common goal, these solutions have unique characteristics, which could allow diversification in robustness, speed, and complexity, among others. Regarding SVM, a widely adopted technique in classification, it is essential to highlight its limitations. Despite its ease of use and adaptability, SVM requires a prior feature extraction stage, which can significantly lengthen the total classification time and affect its accuracy compared to RNNs. This work has not only shed light on the potential of recurrent neural networks in EMG data classification but has also pointed out important directions for future research, especially regarding the optimization and adaptability of the models according to the specific requirements of each application.

## 7. Conclusions

In this study, an in-depth analysis has been carried out on the effectiveness of recurrent neural networks, focusing on LSTM, GRU, and bidirectional architectures, for the EMG signal classification task. The applied methodology, complemented by optimizing hyperparameters using the GWO algorithm, has allowed us to achieve outstanding results, reaching 100% precision in the evaluation stage during the first experimental phase. RNNs, compared to traditional SVM models, show greater versatility in handling input data. This advantage is because recurrent neural networks can directly process data sequences after preprocessing, eliminating the need for specific feature extraction. This comparison highlights the advantage of using RNNs for EMG signal analysis, underscoring their ability to capture and learn from temporal sequences in the data, a limitation in models like SVM.

However, when moving to the second experimental phase, a slight decrease in the accuracy of the models was noticed: LSTM obtained 98.46%, GRU 96.38%, and bidirectional 97.63%. Although these results indicate a slight drop in performance, they are still remarkably high and demonstrate the robustness of recurrent neural networks in the task in question. This variation in results can be attributed to differences in training and validation settings between the two experimental phases, as well as the intrinsic nature of the data. It highlights the importance of the careful selection and tuning of hyperparameters specifically tailored to the characteristics of each dataset and stage of the experiment.

Despite the challenges and decreased accuracy observed in the second phase, RNN-based models have proven robust and practical tools for arm movement classification from EMG signals, maintaining outstanding performance throughout the experiment.

## Figures and Tables

**Table 1:** Overview of EMG classification methods in related studies.

Reference	Classification Method	Tuning Algorithm	Dataset	Accuracy (%)	No. of Channels
[16]	Bidirectional Convolutional-GRU	-	Ninapro DB5 Field	88.70	16
[17]	RNN-LSTM	Manual hyperparameter tuning	500 samples from 5 different gestures	87	4
[18]	KNN	ABC improved	Ninapro	97.06	12
[19]	LSTM	-	UC2018 DualMyo and the Ninapro DB5	95 for DualMyo and 91 for Ninapro DB5	16
[20]	LSTM	-	CapMyo	98.57	8 × 16 electrode array
[10]	SVM	PSO	Healthy muscular limbs collection	91.00	4
[21]	LTSM	PSO	Lower limb muscle	98.58	4
[6]	1D CNN-RNN	-	gForce EMG Armband	91	8
[22]	CNN-LSTM	PCA	Codamotion system acquisition	98.50	16

**Table 2:** Action of the muscles whose action potentials are used for movement classification.

Muscle	Action
Biceps brachii (long head)	Flexes the forearm at the elbow joint, supinates the forearm at the radioulnar joints, and flexes the arm at the shoulder joint.
Triceps brachii (long head)	Extends the forearm at the elbow joint and extends the arm at the shoulder joint.
Superficial flexor of the fingers	Flexes the middle phalanx of each finger at the proximal interphalangeal joint, the proximal phalanx of each finger at the metacarpophalangeal joint, and the hand at the wrist joint.
Finger extensor	Extends the distal and middle phalanges of each finger at the interphalangeal joints, the proximal phalanx of each finger at the metacarpophalangeal joint, and the hand at the wrist joint.

**Table 3:** Hyperparameters considered for adjustment using GWO.

Hyperparameter
Number of neurons per layer
Batch size
Training epoch
Learning rate

**Table 4:** Hyperparametervalues optimized using GWO.

Parameter	Considered Range
Number of neurons per layer	10 to 150
Batch size	128 to 512
Training epochs	10 to 100
Learning rate	0.00001 to 0.1

**Table 5:** Hyperparameter values selected using GWO for LSTM, GRU, and bidirectional neural network.

Hyperparameter	LSTM	GRU	Bidirectional
Number of neurons in first layer	28	25	14
Number of neurons in second layer	74	18	13
Batch size	188	329	199
Training epochs	10	21	16
Learning rate	0.00346	0.00554	0.0117

**Table 6:** Training, validation, and prediction times for LSTM, GRU, and bidirectional neural network.

Model	Training Time (s)	Validation Time (s)	Prediction Time (ms)
LSTM	31.47	0.81	0.12
GRU	51.28	0.85	0.134
Bidirectional	81.60	1.23	0.2

**Table 7:** Features selected by the sensor for classification using SVM.

Sensor	Feature
1	Wavelength
2	Log detector
3 and 4	Shannon entropy
2 and 4	Myopulse percentage rate
3	Modified mean absolute value type 1
3	Zero crossings

**Table 8:** Training, prediction times, and testing accuracy for LSTM, GRU, bidirectional neural network, and SVM.

Model	Training Time (s)	Prediction Time (ms)	Accuracy
LSTM	31.47	0.81	100%
GRU	51.28	0.85	100%
Bidirectional	81.60	1.23	100%
SVM	14	2.7	93%

**Table 9:** Classifier performance for the testing step for SVM.

	Class 1 (%)	Class 2 (%)	Class 3 (%)	Class 4 (%)	Class 5 (%)
Sensitivity	85.2	81.9	80.2	63.9	72.1
Specificity	95.6	93.2	95.9	83.2	82.9

**Table 10:** Hyperparameter values for LSTM, GRU, and bidirectional neural network for the second stage.

Hyperparameter	LSTM	GRU	Bidirectional
Number of neurons in first layer	31	22	15
Number of neurons in second layer	13	101	15
Batch size	206	339	206
Training epochs	14	19	15
Learning rate	0.00502	0.00731	0.0177

**Table 11:** Training, validation, and prediction times for LSTM, GRU, and bidirectional neural network for the second stage.

Model	Training Time (s)	Validation Time (s)	Prediction Time (ms)
LSTM	52.76	1	0.21
GRU	57.90	1.2	0.24
Bidirectional	115.16	2.6	0.34

**Table 12:** Accuracy of LSTM, GRU, and bidirectional neural network for testing. The accuracy for the first stage was 100% for all models.

Model	Second Stage Accuracy
LSTM	98.46%
GRU	96.38%
Bidirectional	97.63%

**Table 13:** Sensitivity (sens) and specificity (spec) of LSTM, GRU, and bidirectional models for the different movements.

Model	Class 1		Class 2		Class 3		Class 4		Class 5	
	Sens (%)	Spec (%)	Sens (%)	Spec (%)	Sens (%)	Spec (%)	Sens (%)	Spec (%)	Sens (%)	Spec (%)
LSTM	99.6	99.9	98.5	99.7	99.9	99.9	98.0	99.5	97.5	99.2
GRU	100.0	99.8	98.0	98.3	99.8	100.0	94.6	99.4	91.4	98.3
Bidirectional	98.4	99.7	95.4	99.8	99.0	99.7	98.1	99.0	96.0	98.5

## Data Availability

Data are contained within the article.
